# The Minimum Information about a Molecular Interaction CAusal STatement (MI2CAST)

**DOI:** 10.1093/bioinformatics/btaa622

**Published:** 2020-07-08

**Authors:** Vasundra Touré, Steven Vercruysse, Marcio Luis Acencio, Ruth C Lovering, Sandra Orchard, Glyn Bradley, Cristina Casals-Casas, Claudine Chaouiya, Noemi del-Toro, Åsmund Flobak, Pascale Gaudet, Henning Hermjakob, Charles Tapley Hoyt, Luana Licata, Astrid Lægreid, Christopher J Mungall, Anne Niknejad, Simona Panni, Livia Perfetto, Pablo Porras, Dexter Pratt, Julio Saez-Rodriguez, Denis Thieffry, Paul D Thomas, Dénes Türei, Martin Kuiper

**Affiliations:** Department of Biology, Norwegian University of Science and Technology (NTNU), Trondheim 7491, Norway; Department of Biology, Norwegian University of Science and Technology (NTNU), Trondheim 7491, Norway; Department of Clinical and Molecular Medicine, Norwegian University of Science and Technology (NTNU), Trondheim 7491, Norway; Functional Gene Annotation, Preclinical and Fundamental Science, Institute of Cardiovascular Science, UCL, University College London, London WC1E 6JF, UK; European Molecular Biology Laboratory, European Bioinformatics Institute (EMBL-EBI), Wellcome Genome Campus, Hinxton, Cambridgeshire CB10 1SD, UK; Computational Biology, Functional Genomics, GSK, Stevenage SG1 2NY, UK; Swiss-Prot Group, SIB Swiss Institute of Bioinformatics, 1211 Geneva 4, Switzerland; Aix Marseille Univ, CNRS, Centrale Marseille, I2M Marseille 13331, France; European Molecular Biology Laboratory, European Bioinformatics Institute (EMBL-EBI), Wellcome Genome Campus, Hinxton, Cambridgeshire CB10 1SD, UK; Department of Clinical and Molecular Medicine, Norwegian University of Science and Technology (NTNU), Trondheim 7491, Norway; The Cancer Clinic, St. Olav’s Hospital, Trondheim University Hospital, Trondheim 7030, Norway; SIB Swiss Institute of Bioinformatics, Geneva 1211, Switzerland; European Molecular Biology Laboratory, European Bioinformatics Institute (EMBL-EBI), Wellcome Genome Campus, Hinxton, Cambridgeshire CB10 1SD, UK; Enveda Therapeutics, 53225 Bonn, Germany; Department of Biology, University of Rome Tor Vergata, Via della Ricerca Scientifica, 00133 Rome, Italy; Department of Clinical and Molecular Medicine, Norwegian University of Science and Technology (NTNU), Trondheim 7491, Norway; Division of Environmental Genomics and Systems Biology, Lawrence Berkeley National Laboratory, Berkeley, CA 94720, USA; Vital-IT Group, SIB Swiss Institute of Bioinformatics, Quartier Sorge, Amphipole Building, 1015 Lausanne, Switzerland; Department of Biology, Ecology and Earth Sciences, University of Calabria, Ecology and Earth Science, Via Pietro Bucci Cubo 6/C, Rende 87036, CS, Italy; European Molecular Biology Laboratory, European Bioinformatics Institute (EMBL-EBI), Wellcome Genome Campus, Hinxton, Cambridgeshire CB10 1SD, UK; European Molecular Biology Laboratory, European Bioinformatics Institute (EMBL-EBI), Wellcome Genome Campus, Hinxton, Cambridgeshire CB10 1SD, UK; Department of Medicine, University of California San Diego, La Jolla, CA 92093, USA; Institute of Computational Biomedicine, Heidelberg University, Faculty of Medicine, 69120 Heidelberg, Germany; Joint Research Centre for Computational Biomedicine (JRC-COMBINE), Faculty of Medicine, RWTH Aachen University, Aachen 52062, Germany; Institut de Biologie de l’ENS (IBENS), Département de Biologie, École Normale Supérieure, CNRS, INSERM, Université PSL, 75005 Paris, France; Division of Bioinformatics, Department of Preventive Medicine, University of Southern California, Los Angeles, CA 90007, USA; Joint Research Centre for Computational Biomedicine (JRC-COMBINE), Faculty of Medicine, RWTH Aachen University, Aachen 52062, Germany; Department of Biology, Norwegian University of Science and Technology (NTNU), Trondheim 7491, Norway

## Abstract

**Motivation:**

A large variety of molecular interactions occurs between biomolecular components in cells. When a molecular interaction results in a regulatory effect, exerted by one component onto a downstream component, a so-called ‘causal interaction’ takes place. Causal interactions constitute the building blocks in our understanding of larger regulatory networks in cells. These causal interactions and the biological processes they enable (e.g. gene regulation) need to be described with a careful appreciation of the underlying molecular reactions. A proper description of this information enables archiving, sharing and reuse by humans and for automated computational processing. Various representations of causal relationships between biological components are currently used in a variety of resources.

**Results:**

Here, we propose a checklist that accommodates current representations, called the Minimum Information about a Molecular Interaction CAusal STatement (MI2CAST). This checklist defines both the required core information, as well as a comprehensive set of other contextual details valuable to the end user and relevant for reusing and reproducing causal molecular interaction information. The MI2CAST checklist can be used as reporting guidelines when annotating and curating causal statements, while fostering uniformity and interoperability of the data across resources.

**Availability and implementation:**

The checklist together with examples is accessible at https://github.com/MI2CAST/MI2CAST

**Supplementary information:**

Supplementary data are available at *Bioinformatics* online.

## 1. Introduction

Causal interactions describe interacting biomolecules involved in processes where the state of one biomolecule is affected by the state of another biomolecule. A formal description of such causal interactions is referred to as a causal statement. A causal statement describes a binary interaction between two biological entities (e.g. gene, protein and RNA), where, given a certain context, the action of a source entity (i.e. the regulator) influences the activity (either directly or by affecting the quantity) of a target entity, which itself may have an altered influence on further downstream targets. For instance, the protein LYN phosphorylates PTPN6 at the C-terminal Tyr-564 site, stimulating PTPN6’s tyrosine phosphatase activity (Yoshida *et al.*, 1999). In other words, the kinase activity of LYN (source entity in an active state) can cause an increase in the phosphatase activity of PTPN6 (change of state of target entity). Additional aspects relating to the when, where and how of the causal interaction are important elements that together capture the context in which this causal interaction occurs (e.g. taxon, cell type and experimental condition). 

The Proteomics Standards Initiative Molecular Interaction (PSI-MI) community was initially driven by the need to curate undirected molecular interactions (Deutsch *et al.*, 2017; Hermjakob, 2006). Yet, since most physical interactions are known to be involved in regulatory processes, several knowledge bases started to collect causal interactions by incorporating directionality information as well (Fazekas *et al.*, 2013; Perfetto *et al.*, 2016; Türei *et al.*, 2016). Therefore, the PSI-MI standard has been extended to also represent the causality of interactions through a direction and sign (up- or down-regulation) (Perfetto *et al.*, 2019). In parallel, the Gene Ontology [GO (Ashburner *et al.*, 2000)], since 2003, has included a ‘regulation of biological process’ (GO:0050789) branch that has been widely used to annotate causal interactions (Balakrishnan *et al.*, 2013), and has recently been extended into the GO Causal Activity Models (GO-CAM) framework (Thomas *et al.*, 2019). The extraction and annotation of causal interactions are predominantly performed via detailed manual curation of scientific publications (Perfetto *et al.*, 2016); but as techniques to infer causality through natural language processing (Todorov *et al.*, 2019) or omics data using prior knowledge (Babur *et al.*, 2018; Bradley and Barrett, 2017; Chindelevitch *et al.*, 2012) are maturing, their results should also be supplied with essential context details. Current formats of causal statements range from the simplest, with only two entities and the causal relationship [e.g. the Simple Interaction Format (SIF) with ‘A activates B’ or ‘A -> B’], to more complex statements including contextual description [e.g. BEL (Biological Expression Language) (Hoyt *et al.*, 2018; Slater, 2014), GO-CAM (Thomas *et al.*, 2019) and PSI-MITAB2.8 (Perfetto *et al.*, 2019)]. At present, various resources cover molecular causal relationships [e.g. IntAct (Orchard *et al.*, 2014), SIGNOR (Licata *et al.*, 2020; Perfetto *et al.*, 2016), Causal Biological Network (Boué *et al.*, 2015), SignaLink (Fazekas *et al.*, 2013), TRRUST (Han *et al.*, 2018), TFacTS (Essaghir *et al.*, 2010) and DoRothEA (Garcia-Alonso *et al.*, 2019)], each adhering to some of the formats mentioned above and annotated with specific controlled vocabularies (CVs) or ontologies [PSI-MI CV , GO]. However, the contextual information provided in different resources can be depicted using different nomenclatures, or be incomplete or inconsistent, resulting in incompatibilities or conflicting information that hinders data integration and can complicate network building (Türei *et al.*, 2016). For example, entity A can be annotated to activate entity B in one database and inhibit entity B in another. Causal statements expressing these seemingly conflicting events are not necessarily incorrect, provided that there is sufficient context description to distinguish when each case occurs. A first step to improve the description of these interactions and their regulatory context is to standardize the different pieces of information and assemble them in a checklist. By adequately annotating and archiving the necessary and sufficient details, causal interactions can be efficiently shared and processed with computers (e.g. for regulatory network assembly) and humans alike (e.g. for designing experiments).

In response to the ‘reproducibility crisis’ in science (Baker, 2016), novel projects focus on setting up formal structures for data management with collaborations between domain experts (Dräger and Palsson, 2014; Mayer, 2009; National Academies of Sciences *et al.*, 2019). For instance, the description of molecular interactions has been formalized by the Human Proteome Organization (HUPO) PSI-MI community (Hermjakob *et al.*, 2004), leading to standard guidelines [MIMIx (Orchard *et al.*, 2007)], exchange formats [PSI-MI TAB (Perfetto *et al.*, 2019), PSI-MI XML (Sivade *et al.*, 2018)] and CVs [PSI-MI CV (Orchard *et al.*, 2005)]. These standards are adopted by biological databases [e.g. IntAct (Orchard *et al.*, 2014), SIGNOR (Licata *et al.*, 2019; Perfetto *et al.*, 2016) and Reactome (Fabregat *et al.*, 2018)], and researchers are called upon to describe their data following these standards (Tripathi *et al.*, 2016). Developing a standardized framework for specific fields increases interoperability between resources (Dräger and Palsson, 2014; Stanford *et al.*, 2015) and helps to improve data findability, reuse and reproducibility. Ontologies and CVs foster unambiguous semantics for the data, underpinned by unique identifiers [e.g. the Gene Ontology (Ashburner *et al.*, 2000; The Gene Ontology Consortium, 2019)], as their terms are used in annotation processes to attribute information to biological entities. Checklists with contextual details to be included in the description of data have been developed [e.g. MIAME (Brazma *et al.*, 2001), MIMIx (Orchard *et al.*, 2007)] and form a fundamental basis for the development of guidelines (Taylor *et al.*, 2008). When semantics and checklists have been agreed upon, standard formats can be built for syntactic support, enabling the storage and exchange of information. The corresponding annotation guidelines advise the curators on the steps and necessary fields to complete to deliver valuable data. Finally, tools ranging from annotation tools to third-party software that can read, write and validate files, endorse these guidelines and formats.

What was missing until now was an authoritative checklist of minimum standards and best practices for annotating causal interactions, building as much as possible on existing sets of standards developed by different communities. A shared standard also provides an integrative framework that allows the mapping of metadata between various resources and enhances data interoperability. We define here the Minimum Information about a Molecular Interaction CAusal STatement (MI2CAST), as a foundation for a formal, consistent and intelligible data capture of causal interactions in molecular biology. It is developed to accommodate the needs of a data user, while considering the practical experience from biological curators. MI2CAST considers terms used in formats mentioned previously (e.g. PSI-MITAB2.8, BEL and GO-CAM) and covers the full range of metadata that should ideally be annotated during the curation process to enrich the description of a molecular causal interaction. MI2CAST checklist advises: (i) the molecular biologists to experimentally assess and describe a list of criteria, when conducting experiments, necessary to contextualize causal interactions; (ii) the curators to consider and extract a list of metadata while curating causal interactions; (iii) the data consumers to access persistent information and fully contextualized data to be able to select causal statements that comply with the system analyzed in their case study. These guidelines do not dictate the format in which one should represent causality, but rather guide on concepts that should be archived together with the causal interaction. Complying with these guidelines should be considered as good practice for the annotation of causal statements to generate high-quality statements.

## 2. The Minimum Information about a Molecular Interaction Causal Statement (MI2CAST)

**Fig. 1.**
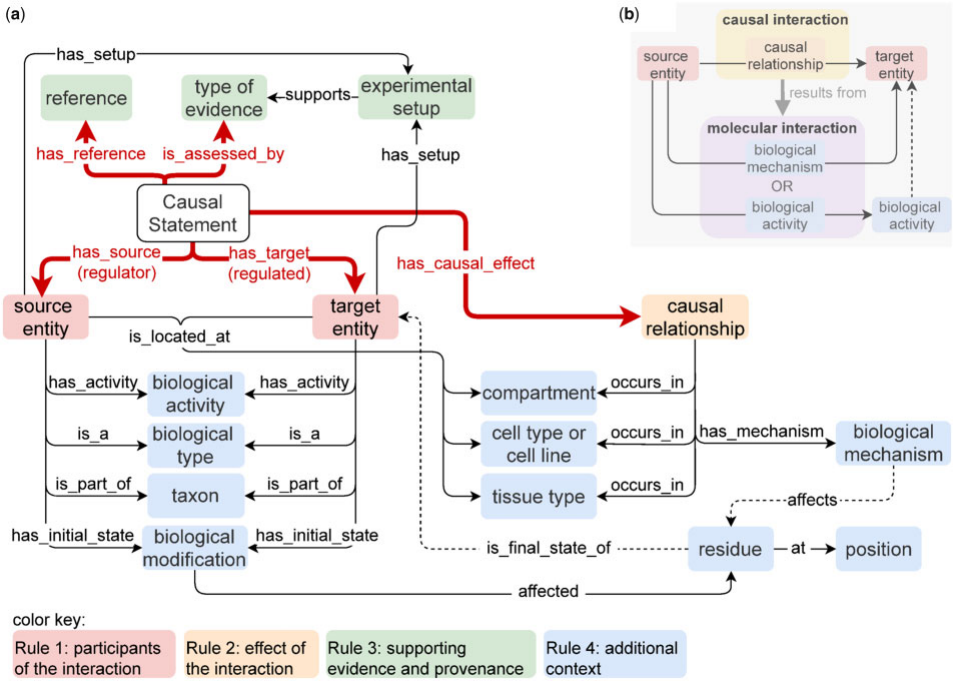
**Data structure diagram documenting the causal statement terms and their relationships.** (**a**) Red thick arrows represent the minimal and mandatory annotations about a causal statement: the source entity, the target entity and the causal relationship of the interaction (red and orange boxes belonging to Rules 1 and 2, respectively), as well as the provenance of the causal statement (green boxes belonging to Rule 3). The black arrows correspond to useful but optional annotations about the entities or causal relationship (the blue boxes belonging to Rule 4). The dotted arrows highlight that when the biological mechanism ‘affects’ a residue, the residue in question is a modification that specifies the final state of the target entity. (**b**) A causal relationship, or ‘causal effect’, between two entities is the result of an associated ‘molecular interaction’ between them, which is specified through either a mechanism or the activity of the source entity (see Rule 4.1)

The MI2CAST checklist structures the information describing the causality associated with a molecular interaction (Fig. 1, see also Supplementary File S1). There is not one single way to represent causal statements, but different alternatives should share a core of mutually compliant information. Their representation depends on the research interest, the available knowledge and specific use cases. For instance, the molecular biologist might be interested in the fine details of the mechanistic events that lead to the expression of a gene (e.g. epigenetic modifications), while a modeler may be interested only in the resulting activation changes (e.g. signaling cascade of interactions between proteins) in addition to the metadata that helps to assess the strength of the evidence. The purpose of MI2CAST is to support and increase the compatibility of these different representations. In addition, a minimum level of context description seems essential for any subsequent reuse of annotated causal interactions. The MI2CAST guidelines lay out these annotation tasks in four rules covering different aspects of a causal interaction (Fig. 1). Each rule specifies terms corresponding to the metadata to annotate, for which recommendations on ontologies and CVs to use are included. When possible, the annotation with a specific piece of information should always use the lowest possible level (i.e. most specific) term from the ontologies or CVs. Rule 1, Rule 2 and Rule 3 cover the most essential information, while Rule 4 recommends annotation of additional details that increases the information content of a causal statement. Note that, different instances of a causal interaction should be provided when the context is different, even if the involved entities are the same.

The MI2CAST guidelines are structured into four rules.

## Rule 1: the source and target entities must be specified

All molecular interaction causal statements must provide reference identifiers of at least a source entity and a target entity. The source entity corresponds to the upstream entity of a causal statement and controls the state (activity or quantity) of the target entity. The target entity corresponds to the regulated entity of a causal statement and is controlled by the source entity. The direction of the interaction is specified: the molecular state change is exerted by the source entity and affects the target entity. For a causal interaction to occur, it is assumed that the annotated context about the source entity (see Rule 4 below) specifies a set of additional circumstances under which the target entity is affected. The source and target can be any molecular entity, for instance a protein, although in reality, molecular entities may not always refer to individual physical entities but rather to populations of individuals of a specific class of molecules: when it is stated that A regulates B in context C, it is actually a population of A that regulates the size or activity of the population of B, in context C. In addition, entities other than biomolecules may also be sources or targets in a causal statement. This enables annotation of the causal relationship that a biomolecule exerts on an observable phenomenon (e.g. a phenotype-like DNA repair or apoptosis), or vice versa. Causally relating a biomolecule and a phenotype is abundantly used in biology, as it enables: (i) to capture knowledge about a process when the curator lacks information regarding downstream molecular events, (ii) to more easily assess the phenotypic outcome of a signaling network (e.g. cell survives, apoptosis is activated) during analysis and (iii) to highlight relevant paths of information flow where mechanistic details may otherwise remain implicit in dense signaling networks. MI2CAST also specifies how to capture relevant context, but for molecular entities only; and its Rules 3 and 4 below apply only to biomolecules.

**Fig. 2.**
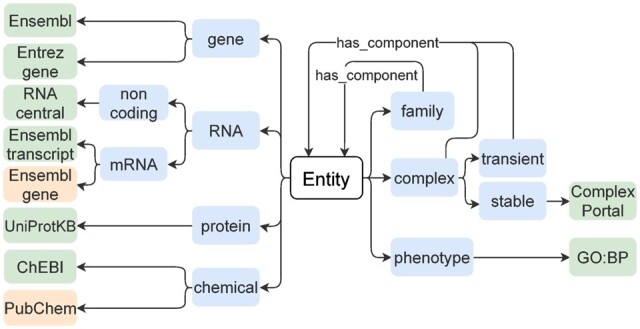
Diagram of entity types and related databases for identifier origin. Blue boxes show the different entity types; green boxes, primarily recommended databases and orange boxes, alternative databases (see also Supplementary File S3). Identifiers from specific databases are recommended for each entity type.

An exhaustive list of entity classes that can be part of causal statements is provided in Figure 2, together with recommendations of comprehensive and widely used ontologies and CVs to describe them. For instance, if the source entity is a known ‘mRNA’, it is recommended to use an ‘Ensembl transcript’ identifier. If the exact mRNA entity is not known, the ‘Ensembl gene’ identifier should be provided, and the ‘biological type’ of the entity (see Rule 4.2 below) must be specified (e.g. ribonucleic acid, messenger RNA). For chemicals that do not have a ChEBI identifier, a PubChem identifier would be an alternative. When the entity is a protein, it can often be present in different isoforms. If the isoform is known, it is recommended to provide the UniProtKB isoform accession number, otherwise the generic UniProt identifier. In addition, it is recommended to annotate a protein with a UniProtKB/Swiss-Prot-reviewed identifier, when available, instead of a UniProtKB/TrEMBL (i.e. unreviewed) identifier. In the case of a ‘family’ (i.e. group of entities with similar functions, sequence or structure) or a ‘transient complex’ [i.e. group of entities that interact together temporarily (Acuner Ozbabacan *et al.*, 2011)], the list of individual entities should be provided (e.g. if a complex has proteins as components, one should provide UniProtKB identifiers for the components of the complex). To be able to distinguish between a complex and a family, the ‘biological type’ of the entity must be provided (see Rule 4.2 below). The phenotype is a distinct type of entity that refers to biological processes associated with molecular events (e.g. TP53 activates apoptosis). This list does not preclude the use of other identifiers (see Supplementary File S3 for a more extensive list of identifiers), as long as appropriate ones are provided.

## Rule 2: the causal relationship of the interaction must be specified

All causal statements must provide the regulatory effect of the molecular interaction. This relationship describes the causal nature of the interaction between the source and the target. It should preferably include the regulatory outcome exerted by the source on the target, i.e. positive or negative, if known. It can also specify whether the interaction is direct or indirect. A direct interaction involves physical contact between the entities. An indirect interaction implies that source and target are not necessarily in direct contact; e.g. the causation could be mediated by intermediate steps that are not explicitly specified. For instance, when a transcription factor positively regulates a protein via transcription, it is an indirect interaction because the transcription factor acts on a gene to produce the protein. In general, the causal relationship implies an increase or decrease in a particular activity of the target, which will affect the process that this target is involved in. It is recommended that one of the following ontologies, and CVs are used to annotate the causal relationship information:

- the ‘causally related to’ branch of the Relation Ontology (RO 0002410), which offers a wide spectrum of annotation of causal relationships,
- the ‘causal statement’ branch of PSI-MI (MI:2233).

When evidence or knowledge of a physical association mechanism is available, or the regulatory outcome is known, curators should use the most specific term from the ontology or CV that is justified by the experimental evidence.

## Rule 3: the provenance and evidence types of the annotation must be specified

A basic task in any annotation procedure is to keep track of provenance (i.e. reference to scientific reports), as it allows consumers of a causal statement to check the quality of an annotation, and the supporting evidence. This evidence may either be curated from biological or other assays, or acquired from computational analysis. Provenance and evidence types affect trust that a data user may have and influence the decision of incorporating a causal statement in a model. For instance, one may consider a manually curated statement more valuable and trustworthy than an automatically generated one because of the errors that may be associated with computational inference and text-mining extraction of causal interactions (Britan *et al.*, 2018).

### 3.1 The reference

When a causal statement is curated manually from an experiment, it is always extracted from a description of that experiment, usually a publication, so the reference to that publication or other source must be provided. If a combination of several articles has led to the finding of a causal interaction, then the full list of these publications must be provided. Each of the publications in the list provides a necessary but not sufficient part of the evidence, and the full list is a minimal set of articles that provide sufficient evidence to support the causal interaction. For instance, if two studies (1 and 2) of a causal molecular interaction performed within the same biological context report (1) a causal effect between two molecules and (2) a physical binding of these same molecules, one may infer by induction from both that there is a direct causal effect involving these molecules. Statement trustworthiness should not be assessed by counting references within a statement. Still, multiple statements expressing the same causal interaction, but each with different reference(s), could make it more trustworthy: the fact that a specific causal relationship is observed in multiple independent experiments by independent researchers may be indicative of the reproducibility of experimental conditions under which the causal interaction occurs. It is recommended that the PubMed identifier is used for the article(s) curated. A Digital Object Identifier (DOI) can also be provided in case of articles not referenced in MEDLINE, e.g. to refer to manuscripts available in preprint servers.

### 3.2 The type of evidence

The type of evidence for declaring and annotating a causal statement must be provided. This information corresponds to the experimental or other data on which the causal interaction is based. The causal statement may be electronically inferred (e.g. through text-mining or *in silico* study), observed during a certain experiment (in which case *in vivo* and *in vitro* studies can be specified), just mentioned (author statement) in a paper, or based on a combination of evidence as in Rule 3.1 (e.g. a causal interaction assessed from the necessary combination of an author statement and the results of an experiment). In the latter case, multiple identifiers can be recorded. When the evidence type is an experiment, the annotation can either be at the less specific level (e.g. experimental evidence, ECO:0000006) or as specific as possible (e.g. a yeast 2-hybrid evidence used in manual assertion, ECO:0005805). The type of evidence should be specified with terms from the Evidence & Conclusion Ontology [ECO (Giglio *et al.*, 2019)].

### 3.3 The experimental setup

If the type of evidence is an experiment (Rule 3.2), the particular experimental conditions that support the causal statement should be recorded, to enable users to select causal statements that meet a particular confidence level. An experimental setup can also be used to specify metadata about the source and target (i.e. the set of experimental procedures used to construct, produce, purify, etc.). For example, if the type of evidence for a causal statement is a ‘reporter gene assay’, additional metadata could be added to the entities, e.g. the source entity was overexpressed (MI:0506), and both the source and the target entities were engineered (MI:0331). The following recommended ontology/CV should be used to capture the experimental condition:

- the Evidence & Conclusion Ontology (ECO),
- the PSI-MI ‘experimental preparation’ branch (MI:0346),
- the Ontology for Biomedical Investigations [OBI (Bandrowski *et al.*, 2016)].

## Rule 4: the defining contextual details should be specified

While causal statements as they are defined above are already useful for building mechanistic models, their relevance becomes even greater when they indicate the experimental context of the corresponding observation. If applied to any other context, it is possible that the causal interaction does in fact not occur for that context. Defining the contextual details may also help to disambiguate statements that would otherwise appear to be conflicting, because the nature of a causal relationship in a given interaction can vary depending on the context. This information benefits data users, who may need to select relevant causal interactions valid for specific conditions. The better the contextual information is, the lower the chance that the causal statement is taken as generally valid and wrongly applied. All molecular interaction causal statements should therefore provide the biological contextual details that are essential to define the specific circumstances in which the causality has been observed (e.g. interaction observed in a particular cell type). The context can be attributed to the source entity, the target entity or the interaction itself. A comprehensive description of the context in which a causal interaction has been observed is essential for humans and computers to infer knowledge and generate hypotheses. Of course, when a paper does not provide certain contextual details a curator cannot annotate them, but when these details are described, it is highly recommended to include them. The conditions under which the context and particulars are optional or required to be annotated are described in the following sections (Rules 4.1–4.5).

### 4.1 The biological activity of an entity, or the mechanism of an interaction

Whenever it is known, the causal interaction should specify the mechanism by which the source entity affects the target entity. For direct interactions, the mechanism can be specified by the activity of the source entity, e.g. the protein kinase activity of protein A causes an effect on protein B. In addition, because a target entity may have more than one activity, or more than one substrate, it is also recommended to associate it with the activity that is affected by the causal interaction. Together, this allows causal statements such as: ‘A, having kinase activity, regulates B, having DNA binding transcription factor activity’. This information enables translation from an entity-based view (used in causal statements) to an activity-based view [used in GO-CAMs (Thomas *et al.*, 2019)]. We recommend using:

- the Gene Ontology Molecular Function terms for proteins and RNA gene products (Ashburner *et al.*, 2000),
- the ChEBI ‘role’ branch (CHEBI:50906) for chemicals (for roles that correspond to a particular activity that the chemical has, e.g. ‘catalyst’),
- the Sequence Ontology for genes (Eilbeck *et al.*, 2005) (for roles of gene features that can be causally affected, e.g. ‘binding_site’).

In the case of indirect mechanisms, or when the molecular activity of the target entity is not known, the mechanism can instead be associated with the entire causal statement. The mechanism describes how the source exerts a biological effect on the target, for instance through a transcriptional regulation. We recommend using:

- the PSI-MI ‘causal regulatory mechanism’ branch (MI:2245),
- the PSI-MI ‘interaction type’ branch (MI:0190),
- the Gene Ontology Biological Process (GO: BP) branch.

Note that for direct interactions, the biological activity of the source entity (e.g. GO molecular function) corresponds to the mechanism of the interaction as specified by a term in the PSI-MI ‘direct interaction’ branch (MI:0407), so these are interchangeable. For example, ‘A, having kinase activity (GO:0016301), regulates B’ corresponds to ‘A regulates, through a phosphorylation reaction (MI:0217), B’. The biological activity of the target entity specifies what function is affected as a result of activity of the source or mechanism.

However, annotating only the biological mechanism of an interaction does not necessarily properly describe its impact on the target entity. Therefore, when the biological mechanism results in a modification (or state-change) of the target (e.g. a phosphorylation event), it is recommended to annotate as precisely as possible how it modifies the target (e.g. with both residue type and position) so as to capture information about the state of the target entity that results from the causal regulation (see also Rule 4.3). If available, not only the end state of the target entity should be captured, but also its affected activity, as described above.

### 4.2 The biological type of an entity

In MI2CAST, the biological type of an entity corresponds to its biological nature, such as gene, RNA, protein and complex. The biological type of an entity is usually defined indirectly, by the identifier provided by the database that aims to list all entities of a certain type (see Supplementary File S3). In most cases, it is therefore not needed to further define the biological type. For instance, a UniProt identifier classifies an entity as a protein. In some cases, however, the biological entity involved in a causal interaction may not yet have a unique identifier assigned to it (see preferred database IDs, Rule 1). We would like to encourage users to contact the appropriate database maintainers and work with them to add that entity. A second option is to search for another database for a gene or gene product that is related to the intended entity, as this will at least allow the use of an identifier rather than make no annotation at all. For example, in the absence of a corresponding UniProt ID, an Entrez ID could be used to annotate a protein. In these cases, the correct and intended biological type of the entity must be provided. In the example, the Entrez ID would have to be accompanied by ‘has biological type: protein’ to clarify that actually the associated gene product is meant. Likewise, when a complex is not referenced in the Complex Portal database (Meldal *et al.*, 2019), it can be specified as a general entity that has a list of components, but should then be annotated with the ‘complex’ (MI:0314) biological type. For biological type, we recommend to use the terms provided by the PSI-MI ‘interactor type’ branch (MI:0313).

### 4.3 The biological modification of an entity

A causality may depend on an entity (source and/or target) having a particular physical modification or conformation prior to its engagement in the causal interaction. Modifications include physical configurations (e.g. post-transcriptional modifications, post-translational modifications, covalent links to other molecules and methylations of genes) that lead to conformational changes (e.g. open and closed) necessary for a causal interaction to occur. If the causality depends on an entity having a particular biological modification, then that state ideally is provided with as much precision as available, and represented by:

- a modification type (e.g. phosphorylation of a protein and methylation of a gene or RNA), specified by PSI-MOD for proteins (Montecchi-Palazzi *et al.*, 2008) and the Sequence Ontology for genes,
- a modified residue (i.e. amino acid and nucleotide), for which we recommend using ChEBI,
- a number indicating the protein sequence position of the residue that is modified.

### 4.4 The taxon of an entity or interaction

For both the source and target entity, the taxon is usually defined through its identifier (e.g. UniProt ID and Ensembl ID). In the case of heterologous system assays, each entity can be annotated with its species of origin. It may be useful for a data user to select causal statements based on taxon ID of the interaction as well. However, as MI2CAST focuses on knowledge that is captured by curation, only the entities’ taxon information needs to be annotated. Of course, any data exchange format based on MI2CAST can still require the inclusion of the taxon at the interaction level. A taxon for the causal interaction as a whole would correspond to the organism in which the interaction has its ‘native function’. For example, if the observed molecular interaction takes place between a source and target entity of the same taxon, then the causal interaction’s taxon would be inferred as being the same. Alternatively, if a causality was observed via an assay in which source and target originate from different taxa (i.e. a heterologous assay), then based on entity homology the causal statement could be computationally inferred as to be valid for both taxa. An identifier from the NCBI Taxonomy (Federhen, 2012) is recommended to capture the taxon.

### 4.5 The location of an interaction or entity

The annotations of physical location specify the precise localization where a causal interaction was observed or where an entity was located. We define different levels of locational definitions, from the highest level being the tissue (Rule 4.5.1) to the most detailed level being the cellular compartment (Rule 4.5.3).

#### 4.5.1 The tissue type

If the tissue type in which the causal interaction has been observed is known, an established ontology identifier should be provided. A tissue type can be annotated at the interaction level or at the entity level, in cases where entities are located in different tissues. BRENDA (Gremse *et al.*, 2011) or Uberon (Mungall *et al.*, 2012) are recommended to capture the tissue type for metazoans, the Plant Ontology [PO (Cooper *et al.*, 2013)] for plants, and the Fungal Anatomy Ontology [FAO (http://purl.obolibrary.org/obo/fao.owl)] for fungi.

#### 4.5.2 The cell type or cell line

If known, the cell type or cell line in which the causal interaction occurs should be provided. A cell type or cell line can be annotated at the interaction level or at the entity level, in cases where entities are located in different cell types or cell lines. The Cell Ontology [CL (Bard *et al.*, 2005; Diehl *et al.*, 2011)] or BRENDA are recommended to capture the cell type. The Cellosaurus (Bairoch, 2018) or BRENDA are recommended to specify the cell line.

#### 4.5.3 The compartment

If the causal interaction is specifically observed in a particular cellular compartment, this should be annotated. The compartment corresponds to the cellular localization where the causal interaction takes place. A compartment can be annotated at the interaction level or at the entity level, in cases where entities are located in different compartments. The interaction can involve multiple compartments (e.g. transport of entities). When the causal statement describes the translocation of a target entity into another compartment, the entity’s original location should be annotated. The entity’s new location could be conveyed by a translocation mechanism term [Rule 4.1; e.g. ‘import into nucleus’ (GO:0051170)]. The terms provided by the Gene Ontology Cellular Component (GO: CC) (Ashburner *et al.*, 2000) are recommended for cellular location annotations.

## 3. Conclusion

MI2CAST describes the Minimum Information about a Molecular Interaction Causal Statement, consisting of a checklist of terms and identifiers recommended for annotations. It takes the form of a set of rules that serve as annotation guidelines. A causal interaction consists of compulsory information on the source entity, the target entity (Rule 1) and the causal relationship (Rule 2). The evidence supporting a causal interaction and its provenance (Rule 3) must also be reported. Annotations describing the defining context of a causal interaction (Rule 4) specify the conditions under which a causal interaction has been observed, together with more detailed information regarding its source entity, target entity and causal relationship. The MI2CAST guidelines have been developed in close collaboration with the GREEKC consortium (greekc.org) and the HUPO Proteomics Standards Initiative (HUPO-PSI) Molecular Interactions workgroup. PSI-MITAB2.8 has been specifically designed to hold MI2CAST-compliant data, enabling the capture of both sign and causality of an interaction. Interestingly, the SIGNOR database already compiles data pertaining to causal relationships between biological entities available in the PSI-MITAB2.8 format. Users will be able to access and merge these data using the MITAB2.8-compliant PSICQUIC webservice (del Toro *et al.*, 2013). Supplementary File S2 provides the compliance to MI2CAST of several formats (SIF, PSI-MITAB2.8, BEL and GO-CAM). The addition of new terms to relevant CVs, such as PSI-MI and Sequence Ontology, as part of the development of these data standards, will enable a fuller description of the biological activity of an entity. MI2CAST remains dynamic, contingent on research insights, requests and the evolution of scientific discoveries in the field of molecular and systems biology. Future extensions could include the recording of logical operators; or the presence of single nucleotide polymorphisms (SNPs) or other variants, which can influence the biological state of entities, and thereby their causal interaction and possible relation to disease states; or the consideration of cells as valid entities for the annotation of cell-to-cell causal interactions (i.e. causality where neither entity is a biomolecule). In summary, MI2CAST represents a next step in the global efforts to take care of valuable life science information.

## Supplementary Material

btaa622_Supplementary_Data
