# Supplementary material for: The Minimum Information about a Molecular Interaction CAusal STatement (MI2CAST)
Source: Bioinformatics. 2020 Jul 8;36(24):5712–8. doi: 10.1093/bioinformatics/btaa622 (PMC8023674; doi:10.1093/bioinformatics/btaa622)
Supplement: btaa622_Supplementary_Data [file btaa622_supplementary_data.pdf]

Supplementary files to the paper: The Minimum Information about a Molecular Interaction Causal Statement (MI2CAST)

Supplementary file S1: Summary table of terms recommended by MI2CAST for the annotation of causal interactions.

Each rule (column 1) in MI2CAST defines specific terms (column 2) to be curated. A definition (column 3) of these terms is given, together with recommended ontologies and CVs (column 4) to use. The 'Required annotation' (column 5) indicates whether the annotation is mandatory or in which cases it is expected to be annotated. Abbreviations are clarified in the main text.

Rule	Term	Definition	Recommended CV/Ontology	Required annotation
1	source entity	regulator entity	see supplementary file S3 below	mandatory
	target entity	regulated entity		mandatory
2	causal relationship	causal regulatory relationship (effect)	MI, RO	mandatory
3	reference(s)	publication(s) where the causal interaction has been curated from	PubMed, DOI	mandatory
	type of evidence	assessment of the causal interaction	ECO	mandatory
	experimental setup(s)	particular experimental setting(s) applied to an entity or the experiment to observe the causal interaction	ECO, MI, OBI	if relevant and described
4	biological activity	molecular function of an entity involved in the causal interaction	GO:MF	if known
	biological mechanism	biological process or interaction type of the causal interaction	MI	if known, and biol. activity is unknown
	biological type	biological nature of the entity.	MI	if ambiguous or incorrect entity's identifier
	taxon	taxon that an entity belongs to, or where the causal interaction is observed	NCBI Taxonomy	if it can not be inferred
	biological modification(s)	physical configuration(s) or conformation(s) of an entity	see documentation	if any, and known
	compartment	cellular location where the causal interaction is observed or an entity is present	GO:CC	if known
	tissue type	tissue structure in which the causal interaction is observed	BRENDA, Uberon, PO, FAO	if known
	cell line or cell type	cell culture or form where the causal interaction is observed or occurs	CL, BRENDA, Cellosaurus	if known

Supplementary file S2: Compliance of existing data formats (mentioned in the Background section) with MI2CAST.

The compliance of the SIF, PSI-MITAB2.8, GO-CAM, and BEL formats is presented in the table below.

For each of the MI2CAST concepts, or terms, (column 1) the status in the four formats is indicated. For SIF (column 2), the '+' sign means that the term can be represented in that format. For PSI-MITAB2.8 (column 3), the MITAB column number(s) indicate where the term can be annotated and stored. The mention '*(work in progress)*' indicates that the term is currently not supported but that the PSI-MI community is working towards improving the compliance of PSI-MITAB2.8 with MI2CAST. Once the formats are updated, the new version of this table will be made available in MI2CAST's GitHub repository (https://github.com/MI2CAST/MI2CAST). More information can be found at: https://psicquic.github.io/MITAB28Format.html.

For BEL (column 4), the table shows how the concepts defined in MI2CAST are handled in the BEL statements and annotations. The annotation of the 'causal relationship' of a causal interaction does not follow the ontologies/controlled vocabularies recommended in MI2CAST to be used. BEL uses symbols, e.g., -> (increases), => (directly increases), -| (decreases), and =| (directly decreases), to indicate an up-regulation or down-regulation. Additionally, BEL also tags the target entity either as the abundance or the activity. For example, this allows for the difference to be made between activating a protein or increasing its expression. More information can be found at: https://biological-expression-language.github.io and https://language.bel.bio.

For GO-CAM (column 5), information about the ontologies used to annotate the different concepts is provided. More information can be found at: http://geneontology.org/cam.

An example of a causal statement annotated following MI2CAST is available in the Supplementary file S4. In addition, other examples are available with different output formats (PSI-MITAB2.8, BEL) at: https://github.com/MI2CAST/MI2CAST/tree/master/examples.

Term	SIF	PSI-MITAB2.8	BEL	GO-CAM
source entity	+	Unique identifier for interactor A (column 1) + Biological role A = 'regulator' (MI:2274, column 17)	(prefix:identifier [! name])	UniProt identifier; MOD (Model Organism Database) gene identifier; Protein Ontology identifier
target entity	+	Unique identifier for interactor B (column 2) + Biological role B = 'regulator target' (MI:2275, column 18)	(prefix:identifier [! name])	UniProt identifier; MOD gene identifier; Protein Ontology identifier

causal relationship	+	Causal statement (column 46)	-> or -| or => or =|	Relation Ontology
reference(s)		Identifier of the publication (column 9)	SET Citation	PubMed identifier or MOD reference identifier
type of evidence		Interaction detection methods (column 7)	SET EvidenceType	Evidence Code Ontology
experimental setup(s)		*(work in progress)*	SET ExperimentalSetupSource, SET ExperimentalSetupTarget	
biological activity		Biological effect of interactor (columns 43, 44)	**act(**(prefix:id), **ma(**prefix:id**))**	GO Molecular Function
biological type		Interactor type (columns 21, 22)	Different possible variations, see BEL documentation.<br><br>Example for protein: proteinAbundance()	
biological mechanism		Causal regulatory mechanism (column 45)	Implicit by combination of biological activity and relationship (see example)	GO Biological Process (for an indirect, “activity regulating” process, e.g. transcription)
taxon		NCBI Taxonomy identifier for interactor (columns 10, 11)	SET Taxonomy	NCBI Taxonomy
biological modification(s)		Feature(s) for interactor (columns 37, 38)	Different possible variations, see BEL documentation.<br><br>Example of protein PTM: p(prefix:identifier, pmod(prefix:identifier))	Protein Ontology (modified proteins)
compartment		*(work in progress)*	SET Compartment	GO Cellular Component
tissue type		*(work in progress)*	SET Tissue	Uberon (animals), Plant Ontology, Fungal Anatomy Ontology, and other MOD-specific ontologies
cell line or cell type		*(work in progress)*	SET CellLine	Cell Ontology, and other MOD-specific ontologies

Supplementary file S3: Extensive list of identifiers that can be used for the annotation of 'entities'.

For each of the entity 'types' (column 1) a definition is provided (column 2), and the ontologies and controlled vocabularies that can be used are shown in column 3. Entries in **bold** are recommended. However, as stated in the main manuscript, these recommendations do not preclude the use of other high quality and maintained ontologies and controlled vocabularies when suited, if appropriate mechanisms to export annotated information to other formats are made available.

Type	Definition	Ontology/Controlled vocabulary
**gene**	A region of a sequence that encodes an RNA transcript.	**Ensembl gene**, **Entrez gene** (NCBI gene), HGNC, MOD (Model Organism Database).
**non-coding RNA**	An RNA sequence that does not translate into a protein.	**RNAcentral**, miRBase, WormBase.
**mRNA**	An RNA sequence that can be translated into a protein.	**Ensembl transcript**, Ensembl gene, MOD.
**protein**	A large biomolecule composed of one or more chains of amino acid residues.	**UniProt**, Protein Ontology.
**chemical**	A small molecule.	**ChEBI**, PubChem, ChEMBL, DrugBank.
**family**	A group of entities that share a common structure and/or function.	List of components, Pfam, PANTHER, InterPro, FamPlex, Protein Ontology.
**transient complex**	A group of entities that temporarily interact together to perform a function (all components are necessary for the complex to perform that function).	List of components.
**stable complex**	A group of entities that permanently interact together to perform a function (all components are necessary for the complex to perform that function).	**Complex Portal**, List of components.
**phenotype**	An observable phenomenon related to a biological process.	**Gene Ontology Biological Process**, HPO (Human Phenotype Ontology), PATO (Phenotype And Trait Ontology), DO (Disease Ontology).

Supplementary file S4: Example of use case that applies the MI2CAST concepts for the manual curation of a causal statement from a publication.

The selected paper for this example is the following: "Characterization of E2F8, a novel E2F-like cell-cycle regulated repressor of E2F-activated transcription" by Christensen *et al.* (2005)[1], that highlights a causal interaction between a transcription factor (E2F8) and a target gene (CCNE1). By following the rules of MI2CAST, it is possible to annotate the following information:

Rule 1: Source and Target entities
uniprot:A0AVK6 (E2F8): the source entity is the E2F8 transcription factor annotated with a UniProt identifier.
ncbigene:898 (CCNE1): the target entity is the CCNE1 gene annotated with an NCBI gene identifier.

Rule 2: Causal relationship
MI:2240 (down-regulates): the causal relationship of the interaction is a down-regulation, annotated with a Molecular Interactions Controlled Vocabulary (MI) term. E2F8 down-regulates CCNE1.

Rule 3:
3.1: Reference
PMID:16179649: the causal statement is assessed in the article entitled "Characterization of E2F8, a novel E2F-like cell-cycle regulated repressor of E2F-activated transcription". The PubMed identifier is given.

3.2: Evidence type
ECO:0005648 (luciferase reporter gene assay evidence used in manual assertion): the observation of the causal interaction is done via a luciferase reporter gene assay experiment and has been assessed by human review. An Evidence and Conclusion Ontology (ECO) identifier is given.

3.2.1: Experimental setup
MI:0506 (over expressed level) and **MI:0331** (engineered) for the source entity: The source entity has been over-expressed and engineered during the experiment, annotated with an MI controlled vocabulary term.
MI:0331 (engineered) and **SO:0001679** (transcription_regulatory_region) for the target entity: the target entity has being engineered and its transcription regulatory region is used during the experiment, annotated with an MI controlled vocabulary term and a Sequence Ontology (SO) term.

---

[1] Christensen,J. *et al.* (2005) Characterization of E2F8, a novel E2F-like cell-cycle regulated repressor of E2F-activated transcription. *Nucleic Acids Res.*, **33**, 5458–5470.

Rule 4:
4.1: Biological activity or mechanism
MI:2247 (transcriptional regulation): the biological mechanism of the causal interaction is a transcriptional regulation, annotated with an MI controlled vocabulary term. E2F8 is a regulator of the transcription of CCNE1.

4.2: Biological type
This is not necessary to be annotated as the correct identifiers have been given: the source entity is a transcription factor (i.e., protein) annotated with a UniProt identifier and the target entity is a gene annotated with an NBCI gene identifier.

4.3: Biological modification
There is no information about a specific biological modification of the source nor the target entity in this article, thus no annotation is added for Rule 4.3.

4.4: Taxon
The taxon information is defined by the entities' identifiers: the UniProt identifier "A0AVK6" is a human E2F8 protein and the NCBI gene identifier "898" is a human CCNE1 gene.

4.5.2: Cell type or cell line
BTO:0001938 (Human osteosarcoma cell line): the causal interaction is observed in the human osteosarcoma cell line.

4.5.3: Cellular component
GO:0005634 (nucleus): the causal interaction occurs in the nucleus and both entities are located in the nucleus, annotated with a gene ontology term.
It can be noted that this information can be inferred from the biological nature of this causal interaction: as the target entity is a gene and the source entity a transcription factor (TF), the TF exerts its influence on the gene inside the nucleus.

More examples can be found at https://github.com/MI2CAST.
